# Cost Analysis of End-Stage Renal Disease in Pediatric Patients in Greece

**DOI:** 10.3390/healthcare12202074

**Published:** 2024-10-18

**Authors:** Christos Ntais, Konstantina Loizou, Costas Panagiotakis, Nikolaos Kontodimopoulos, John Fanourgiakis

**Affiliations:** 1 Epidemiology Program, School of Science & Technology, Hellenic Open University, 26335 Patras, Greece; 2 Healthcare Management Program, School of Social Sciences, Hellenic Open University, 26335 Patras, Greecenkontodi@hua.gr (N.K.); 3 Department of Management Science and Technology, Hellenic Mediterranean University, 72100 Agios Nikolaos, Greece; 4 Department of Economics and Sustainable Development, Harokopio University, 17676 Athens, Greece

**Keywords:** end-stage renal disease, conventional hemodialysis, online hemodiafiltration, peritoneal dialysis, cost analysis, economic evaluation

## Abstract

Background/Objectives: The cost resulting from peritoneal dialysis (PD), conventional hemodialysis (HD) and online hemodiafiltration (OL-HDF) in pediatric patients with end-stage renal disease (ESRD) has not been estimated to date in Greece. The present single-center retrospective study aimed to estimate the mean annual cost of the above methods, as well as the individual components of this cost. Methods: Twenty pediatric patients undergoing the three different methods of renal replacement therapy were included in this study. Their mean total annual cost was estimated by the method of micro-costing and the bottom-up approach. Results: The mean total annual cost for PD patients (n = 7) was estimated at EUR 56,676.04; for conventional HD patients (n = 9), it was EUR 39,786.86; and for OL-HDF patients (n = 4), it was EUR 43,894.73. The PD method was found to be more expensive than the other two methods (*p* < 0.001 vs. conventional HD and *p* = 0.024 vs. OL-HDF). PD consumables used for daily application had the greatest contribution to the total annual cost. The total mean annual cost in the groups of patients undergoing HD and OL-HDF did not differ significantly (*p* = 0.175). The total operating cost of the renal dialysis unit had the greatest contribution to the total mean annual costs of both the conventional HD and OL-HDF techniques. Conclusions: This cost analysis provides useful information to healthcare policymakers who make decisions about the treatment of children with ESRD.

## 1. Introduction

Chronic kidney disease (CKD) is characterized by irreversible functional or structural damage to the kidneys that can lead to chronic end-stage renal disease (ESRD). Although CKD in the pediatric population, compared to adults, is not considered common, its progressive development into ESRD has been associated with reduced somatometric and psychosocial development, poor quality of life, increased mortality and cardiovascular morbidity [1].

Advances in medical devices and the biocompatibility of materials have helped to make dialysis methods easier, safer and more effective, but at a higher cost. Among the dialysis methods, peritoneal dialysis (PD) is a method that has been widely established in the pediatric population, especially in infants and young children, until transplantation is performed [2]. The benefits and advantages of this method are numerous, such as avoiding vascular access and its consequent risks and the lower rate of decline in residual renal function. Despite the increased burden of responsibility on the family and the appropriate education that they must follow in terms of avoiding infections, it is still a home-based treatment that allows children to participate in school and other daily activities [3]. Meanwhile, a smaller number of children around the world undergo the hemodialysis (HD) method. Many of these children switch from the PD method due to peritoneal membrane impairment from recurrent peritonitis, or they are children who, due to intra-abdominal pathologies or social difficulties, are unsuitable for the PD method [4]. Finally, the online hemodiafiltration (OL-HDF) method is a modern HD technique that may have a higher cost but is more effective in clearing higher-molecular-weight substances [4]. Moreover, this method seems to be positively associated with better growth and a lower risk of cardiovascular disease [5]. As a result, it is increasingly used in children, even at younger ages [4,5].

The burden of ESRD on the economy is significant as its prevalence in recent decades has shown a dramatic increase in both adults and children [1,6], thus making its treatment a threat to healthcare resources [7]. Already, in several countries, including Greece, these treatments consume about 2% of the total health expenditure [8,9]. In developing countries, and especially in those where the allocation of health resources to related treatments is inadequate or prohibitive, there is an increase in child mortality from CKD. As a result, the real impact of ESRD on the pediatric population is more severe than what is described by epidemiological data [1].

There are many differences between countries’ health systems in terms of reimbursement for renal replacement therapies. Despite the differences, the common purpose is quality, efficacy and equity in healthcare and resource saving [8,10,11]. To serve this purpose, and under the pressure of constraints with each country’s healthcare budgets, economic evaluations should be conducted to examine all of the cost factors that are involved in ESRD. The cost analysis of the disease helps the relevant stakeholders to formulate healthcare policies, to set priorities and also to properly allocate already finite resources to treatment options [12].

The present study is a single-center retrospective cost analysis study of ESRD in pediatric patients following the three methods of dialysis (PD, conventional HD and OL-HDF) in the Nephrology Department of the General Children’s Hospital “P. & A. Kyriakou” in Athens, Greece. This renal dialysis unit (RDU) is the largest pediatric unit in the country and the only one operating within a pediatric hospital. This study assesses the mean total annual cost of the three methods of dialysis undergone by children in Greece and identifies the factors that contribute to its increase. Estimating the direct and overhead costs, assessing the economic burden, highlighting long-term sustainability issues and identifying inefficiencies and areas for improvement are the purposes of the present study.

## 2. Materials and Methods

### 2.1. Patients

This study includes data from 20 pediatric patients who were followed up with at the Nephrology Department of the General Children’s Hospital “P. & A. Kyriakou” in Athens, Greece, and specifically in the RDU, in the year 2019 (from 1 January 2019 to 31 December 2019). Seven of the 20 patients were treated with PD (automated PD (APD), with 5 of them sometimes also undergoing the technique of continuous ambulatory PD (CAPD)), while 9 were treated with conventional HD and 4 with OL-HDF.

### 2.2. Patient Case Study Form

Three case report forms (CRFs) were created for the purposes of this study, with one for each dialysis method: CRF-A, CRF-B and CRF-C for the PD method, the conventional HD and the OL-HDF, respectively. In order to register data from medical and nursing records, a form was used for each patient undergoing the respective method.

The three case study forms contained eight common sections of questions to collect the following data: registration data (nephrology clinic unit, date of registration, patient and form number); individual patient characteristics (age, date of birth, gender, nationality and insurance provider); individual medical history (cause of CKD and year of diagnosis); data on the initiation of dialysis (first-choice method and date of initiation of dialysis, age of initiation of the method undergone by the patient during the study and the type of surgical preparation/vascular access involved); data collected during the patient’s regular hospital visits (number of HD and OL-HDF visits or sessions per month and somatometric characteristics); the number and type of laboratory tests performed and directly related to the dialysis treatment; medicines and sanitary consumables directly related to the treatment; food for special medical purposes to support the children’s nutrition and development.

### 2.3. Methodology for Determination of Cost

In this cost analysis, the total annual cost for each method of dialysis was calculated. To fully assess and accurately calculate the resources expended, the micro-costing method with a bottom-up approach was used. Micro-costing is a cost estimation methodology employing detailed resource utilization and unit cost data to generate precise estimates of economic costs [13]. The total annual cost is divided into direct medical costs and overhead costs. The direct medical costs are those incurred by the hospital and the insurer, while the overheads are those incurred by the hospital alone.

### 2.4. Calculation of Mean Total Annual Cost of Each Method

The mean total annual cost of the chosen method for each patient included the cost of laboratory tests, medicines (both prescription and hospital drugs), sanitary consumables, food for special medical purposes, allowances (welfare, transport, food), the maintenance of equipment, human resources (salaries of physicians, nurses and ward assistants) and utility costs. In particular, to calculate the cost of laboratory tests, medicines, consumables and food for special medical purposes, the total annual number thereof was multiplied by their unit price. The annual maintenance cost of the equipment included sodium carbonate and citric acid cartridges for cleaning, sterilization and decalcification and microbial ultrafiltration membranes for the conventional HD equipment and thermal disinfection and dialysis solutions, filtration membranes for the purification of transfusion products and citrate solutions for the OL-HDF equipment. The maintenance costs also included the cost of a water treatment system and the costs of spare parts and emergency repairs according to the guidelines provided by the hospital’s Department of Biomedical Technology.

The calculation of staff salaries was based on the 2019 salary scale. The prices of the medicines used were obtained from the official price bulletin of drugs for human use for the year 2019. The prices and consumption of hospital drugs were provided by the hospital pharmacy. The prices of laboratory tests were derived from the listed prices of the corresponding prescriptions based on the related Government Gazette Folio (state tariff). The prices of PD consumables were obtained from the Price Observatory of the Greek Health Procurement Committee, whereas the prices of HD and OL-HDF consumables were provided by the hospital’s supply depot. By summing the total annual costs of all of the above, the total annual cost of each method of dialysis was obtained and then allocated to the patients.

### 2.5. Calculation of Total Annual Hospital Cost

Hospital costs include the cost of medicines billed to the RDU and administered to children orally or intravenously during the HD and OL-HDF sessions (antithrombotic drugs, vitamins, iron and sera) and the cost of the sanitary consumables used, as well as the cost of maintaining the RDU (maintenance of equipment, staff salaries and utilities).

### 2.6. Calculation of Total Annual Cost of Insurance Provider

The cost of the insurance provider included the costs of laboratory tests, prescription drugs, sanitary consumables (peritoneal solutions, filters and solutions of HD and OL-HDF, etc.), allowances and food for special medical purposes.

### 2.7. Statistical Analyses

The demographic and dialysis-related variables of the sample were summarized using descriptive statistics. Categorical data (gender, causes of ESRD, method of dialysis, etc.) are presented as percentages. Cost data are expressed as the mean cost with the standard deviation. An analysis of variance (ANOVA) was performed to compare the overall mean annual costs for each method. All analyses were performed with IBM SPSS Statistics v.24.

## 3. Results

### 3.1. Key Patient Characteristics

Twenty pediatric patients were enrolled in the study, of whom seven (35%) underwent PD, nine (45%) underwent conventional HD and four (20%) underwent OL-HDF. The basic social and demographic characteristics of these patients are summarized in Table 1.

The causes of patients’ ESRD were glomerulopathies in nine patients (45%) and congenital kidney and urinary tract anomalies in eight patients (40%). Other causes were glomerulonephritis (one patient), bilateral renal vein thrombosis (one patient) and a neurogenic cyst with reflux nephropathy (one patient). PD was the first method followed at the start of dialysis in 14 (70%) children. In 89% (eight of nine) of patients undergoing HD and 50% (two of four) undergoing OL-HDF, a central venous catheter (CVC) had been inserted, while the rest (one patient in HD and two patients in OL-HDF) had a fistula.

### 3.2. Total Mean Annual Cost

The mean value for the total annual cost of treatment for PD was found to be EUR 56,676.04; that for HD was EUR 39,786.86; and that for OL-HDF was EUR 43,894.73 (Table 2).

Based on the ANOVA results, the total mean annual cost between PD, HD and OL-HDF differed significantly (*p* < 0.001). The multiple comparison tests performed led to the conclusion that the total mean annual cost in the groups of patients undergoing HD and OL-HDF did not differ significantly (*p* = 0.175), while the cost of the PD method differed significantly from that of HD (*p* < 0.001) and OL-HDF (*p* = 0.024).

### 3.3. Breakdown of Total Mean Annual Cost

Overall, 73.3% of the total mean annual cost of the PD method is due to the consumables required for its daily implementation. This is followed by allowances at 14.3% and then prescription drugs at 6.6%. As for the conventional HD method, the largest contribution to the total mean annual cost is made by allowances (29.2%), the RDU operating costs (25.7%) and prescription drugs (15.1%). Finally, the largest contributions to the total mean annual cost of the OL-HDF method are made by allowances (28.6%), the RDU operating costs (23.3%) and consumables (16.6%) (Table 3).

Regarding prescription drugs, for the PD method, the drugs that contributed the most to the cost were erythropoietin (37.13%), phosphorus binders (34.62%) and growth hormones (21.78%). For the conventional HD method, the cost of medication prescribed to patients was 39.32% for antithrombotic drugs, 27.02% for erythropoietin and 21.93% for growth hormones. Regarding the OL-HDF method, the pharmaceutical cost was mainly due to erythropoietin (32.82%), growth hormones (27.72%) and antithrombotic agents (17.46%).

## 4. Discussion

The present study assesses the costs of the three methods of dialysis followed in a pediatric setting in Greece. The costs derived from this population group have not been estimated to date in this country. As regards the proportion of children who underwent the PD method compared to that of children receiving an artificial kidney (35% vs. 65%), this is in agreement with previous studies in pediatric populations [5,14,15]. Regarding gender, boys outnumbered girls in this study (65% vs. 35%), thus mirroring studies showing that ESRD occurs more frequently in boys [1,6,16]. As far as the causes of CKD are concerned, our study is in agreement with previous epidemiological studies that have identified congenital kidney and urinary tract abnormalities and glomerulopathies as the most common ones [16,17,18,19].

Regarding the cost of the PD and HD methods around the world, after reviewing the relevant literature, Karopadi et al. [20] concluded that PD was the least expensive method in the majority of developed countries. This review included only one cost analysis in a pediatric population. Cullis et al. [21] showcased PD as a more resource- and environmentally efficient treatment with the potential to improve dialysis access, especially for vulnerable populations, including women and children, in lower-resource countries. Furthermore, a cost–utility analysis of the PD- and HD-first policy in Thai adults with ESRD concluded that the PD-first policy still remains more cost-effective than the HD-first policy at the current willingness to pay [22]. However, in the mentioned study, HD yielded more quality-adjusted life years than PD [22]. Coyte et al. [23] estimated the total annual costs of the methods of CAPD, APD and HD in children to be USD 47,669, USD 48,668 and USD 76,023, respectively. The cost difference between PD and HD was due to the cost of maintaining the dialysis unit (by 60%), physicians’ salaries (by 25.8%) and hospital costs caused by complications among HD patients (by 14.2%). In all HD patients, 60% had fistulas and 40% had CVC, whereas, in all PD patients, 35% underwent only CAPD and 65% underwent only APD.

In the present study, it was found that the PD method was more expensive than the conventional HD method and the OL-HDF method, with a total annual cost of EUR 56,676.04, EUR 39,786.86 and EUR 43,894.73, respectively. These results were obtained after a micro-assessment and bottom-up approach using all of the elements that contribute to the costs of the three methods and are borne by the insurance provider and the hospital. However, the costs of treatment complications, such as peritonitis, infections and thrombosis of the catheter and the arteriovenous anastomosis, whose treatment is directly associated with a significant increase in hospital costs, have not been taken into account [24]. Studies have shown that the risk of complications in children who carry a CVC is 11.9%, while, in children undergoing PD, it is 6.3% [25]. The lower risk of peritonitis is confirmed by other studies [24,26], where it has been estimated at one episode every 15 months, while, in contrast, catheter-related complications are estimated at 8.1 episodes per 100 patients annually. Therefore, if the cost of complications were accounted for in this study, there would be a greater burden on the total annual cost of the HD method as 89% (eight out of nine patients) of children had a CVC.

Our study confirms the results of a similar Greek study in an adult population where PD was estimated to be the most expensive dialysis method, with a mean annual cost per patient of EUR 52,057.20, compared to EUR 39,258.50 for HD [27]. In the mentioned study, the majority of adult patients underwent the CAPD method instead of APD (at a rate of 70%–30%, respectively). The rate of using the PD techniques can significantly influence the final cost, since consumables and solutions are the most contributing factors. Indeed, in our study, it was the cost of the solutions and materials necessary to perform PD that contributed the most to the formation of its total annual cost (by 73.3%). It should be noted that, in addition to APD consumables, the majority of the patients in our study, especially those with the loss of residual renal function, also received CAPD consumables to achieve better ultrafiltration. In general, APD is a technique that is widely used in the pediatric population, despite the high cost of its consumables. In addition to the lower likelihood of complications such as peritonitis, being disconnected from the machine during the day enables children undergoing APD to attend school and participate in daily activities [3].

The present study confirms the results of the study by Coyte et al. [23] regarding the significant economic difference found between the PD method and the two techniques of HD due to the RDU operating costs. As the results of our study show, the RDU operating costs, which also included the maintenance of equipment costs, had a huge difference (by 98%) between the two methods. Moreover, these costs were found to have the highest contribution to the total annual cost of the two HD techniques (33.4% and 28.5% for conventional HD and OL-HDF, respectively). These results are also in agreement with the corresponding Greek study in an adult population where a significant economic difference (by 79%) was estimated between the specific costs of the two methods [27].

In addition to the costs of RDU maintenance and operation, PD appears to be superior also in terms of the costs of laboratory tests. In our study, the annual cost regarding the specific expenses of the PD method was found to be lower compared to the conventional HD and OL-HDF methods by 50.7% and 48.7%, respectively. This is reasonable since, in the PD method, laboratory tests are performed monthly and in conjunction with the hospital visit. In contrast, in the two HD techniques, laboratory tests are performed weekly, before and after dialysis.

Regarding the comparison of the conventional HD with the OL-HDF technique, the 18-month cost–benefit study by Ibrahim et al. (2020) in a sample of 34 children (mean age 14.7 years) showed improvements in all biochemical and clinical indicators after the switch from conventional HD to OL-HDF. Among these were the indicators of growth, anemia and adequacy of dialysis. In their study, OL-HDF was found to be 23% more expensive than conventional HD. However, the cost of medication for OL-HDF was found to be lower than that of conventional HD, mainly due to the reduction in the doses of iron, erythropoietin, phosphorus binders and active vitamin D metabolites. In addition, the benefits of each technique were measured by generating benefit scores. Overall, 100% of children undergoing OL-HDF scored positively, compared to only 33% of children undergoing conventional HD. In conclusion, the cost–benefit ratio of OL-HDF was not found to be significantly different from the cost–benefit ratio of conventional HD [28].

In our study, OL-HDF was found to be 9.3% more expensive than conventional HD. Its results are in agreement with the study by Ibrahim et al. [28] regarding the contribution of consumables to the final cost. As shown in Table 3, OL-HDF consumables had almost double the contribution to the total annual cost of OL-HDF compared to conventional HD (16.6% vs. 7.8%, respectively). The additional cost of OL-HDF consumables mainly comes from the high-permeability filters required by this technique and the arteriovenous connection lines.

Regarding medication, in our study, its contribution to the annual cost of PD was much lower compared to conventional HD and OL-HDF (6.6% versus 15.1% and 15.9%, respectively). From the percentage distribution of the total annual cost of medication by drug category, it is evident that erythropoietin administered for the treatment of anemia is the one that contributes the most to the cost of medication for all three methods. Also noteworthy is the difference in the cost contribution of the category of phosphorus-binding drugs administered for the management of hyperphosphatemia. In this category, the costs of two pharmaceutical preparations administered to children with the active substances sevelamer and calcium carbonate were recorded. Sevelamer is a high-cost drug; however, it is effective in managing phosphorus, calcium and PTH levels. Its long-term benefits in terms of calcification and the risk of cardiovascular disease also appear to be significant [29]. However, calcium carbonate is preferred in infants and young children so that the required calcium needs are met and when, due to the young age, there is no compliance with the sevelamer administration scheme [29]. Indeed, in the present study, a significantly lower overall cost burden of phosphorus-binding drugs, and specifically of sevelamer, was observed in the conventional HD method with which patients with a younger mean age were treated, compared to the other two methods.

Transplantation is considered the therapeutic goal of ESDR in children. Given the enormous costs consumed by dialysis methods, cost estimation studies in adults suggest transplantation for younger patients, even with significant comorbidities, as the most cost-effective treatment [30]. Indeed, in a cost analysis study of renal function replacement therapies in children, Camargo et al. [30] compared APD with transplantation. The total monthly cost per patient of APD was found to be USD 3500 and that of transplantation was USD 1900. Based on the results of this study, transplantation emerged as the least expensive treatment compared to any other method of dialysis after the first 13–15 months of the transplantation period.

The sample of this study consisted of 20 patients attending the Nephrology Department of the General Children’s Hospital “P. & A. Kyriakou” in Athens during the year 2019. This unit is the largest pediatric RDU in the country and the only one operating within a pediatric hospital. In addition, according to the official data of the Hellenic Transplant Coordination and Control Service, the total number of children undergoing the PD method in Greece for the year 2019 was 24. Thus, although the small sample size is a weakness of this study, it can be considered that the participating patients were a representative sample of the Greek pediatric population <16 years of age undergoing dialysis during the year 2019.

Another limitation of this study is that the conducted cost analysis utilized the prices prevailing in the Greek healthcare system, rendering its results non-comparable with those in other jurisdictions. For example, in the PD method, the cost of consumables, which counts for 73.3% of the total cost, may differ significantly in other countries. Additionally, social security allowances, which drive the costs in HD and OL-HDF, may also differ significantly in other healthcare systems.

## 5. Conclusions

The present study is the only cost analysis of the three methods of dialysis that pediatric patients undergo in Greece. The PD method is found to be the most expensive, mainly due to the consumables needed for its daily application. However, both the PD and HD methods have complications, which result in additional costs for their treatment. To estimate these additional costs, further research should be carried out in this population group. Moreover, the OL-HDF technique is found to be more expensive than conventional HD. The cost analysis carried out provides useful information to healthcare policymakers who make decisions about the treatment of children with ESRD. In the PD method, it is the consumables and medication that contribute about 80% of the total cost. As for the conventional HD method, which is the one with the lowest cost, it appears that RDU maintenance together with drugs are the factors that contribute about 49% of the total cost. Finally, in the OL-HDF method, consumables contribute significantly to the cost, which, together with RDU maintenance and medicines, account for 61% of the total cost. In conclusion, cost containment policies should focus mainly on all of the above areas, but without the risk of compromising the efficacy, quality and safety of the treatments.

## Figures and Tables

**Table 1:** Basic social and demographic characteristics of the study patients (N = 20).

	PD (N = 7)	HD (N = 9)	OL-HDF (N = 4)	Total (N = 20)
Gender				
Male (%)	4 (57)	7 (78)	2 (50)	13 (65)
Female (%)	3 (43)	2 (22)	2 (50)	7 (35)
Age (years)				
Mean (SD)	9.41 (4.08)	8.20 (3.75)	12.48 (3.30)	10.03 (3.71)
0–4 (%)	1 (14.3)	2 (22.2)	0 (0)	3 (15)
5–9 (%)	2 (28.6)	3 (33.3)	1 (25)	6 (30)
10–14 (%)	4 (57.1)	4 (44.4)	2 (50)	10 (50)
15+ (%)	0 (0)	0 (0)	1 (25)	1 (5)
Nationality				
Greek (%)	5 (71)	9 (100)	3 (75)	17 (85)
Other (%)	2 (29)	0 (0)	1 (25)	3 (15)
Social Security				
EOPYY ^a^ (%)	6 (86)	7 (78)	4 (100)	17 (85)
Uninsured/vulnerable groups (%)	1 (14)	2 (22)	0 (0)	3 (15)

^a^ EOPYY: Greek National Health Services Organisation.

**Table 2:** Total mean annual cost per method of dialysis.

	Total Mean Annual Cost (EUR)	SD (EUR)	Range (EUR)
PD	56,676.04	8332.13	51,005–72,270
HD	39,786.86	4330.92	32,935–43,174
OL-HDF	43,894.73	5619.78	36,285–48,394

**Table 3:** Breakdown of total mean annual costs by dialysis method.

	PD		HD		OL-HDF	
	Mean (SD) (EUR)	%	Mean (SD) (EUR)	%	Mean (SD) (EUR)	%
Laboratory tests	1460.70 (231.96)	2.6	2960.79 (217.10)	7.4	2845.72 (415.26)	6.5
Prescription drugs	3735.54 (2134.43)	6.6	6023.22 (3128.32)	15.1	6964.06 (3049.39)	15.9
Hospital medicines ^a^	0 (0)	0	852.86 (146.03)	2.1	886.90 (225.13)	2.0
Consumables	41,517.09 (6217.70)	73.3	3090.99 (720.51)	7.8	7306.08 (1896.29)	16.6
Other sanitary material	13.67 (3.68)	0	365.96 (61.03)	1.0	280.63 (127.23)	0.6
Maintenance of equipment	0 (0)	0	3044.96 (68.90)	7.7	2254.24 (779.69)	5.1
Unit operating cost	240.00 ^b^ (0)	0.4	10,243.50 (0)	25.7	10,243.50 (0)	23.3
Food for special medical purposes ^c^	1609.02 (2342.15)	2.8	1584.58 (1891.88)	4.0	543.60 (627.70)	1.2
Allowances ^d^	8100.00 (0)	14.3	11,620.00 (2280.00)	29.2	12,570.00 (3420.00)	28.6

^a^ Intravenous treatment administered during the dialysis session and billed to the RDU. ^b^ Relates only to the salaries of the staff of the unit, excluding utility costs. ^c^ Food for special medical purposes was given to 4 of the 7 patients undergoing PD, 5 of the 9 patients undergoing conventional HD and 2 of the 4 patients undergoing OL-HDF. ^d^ Food (EUR 362/month), welfare (EUR 313/month) and transport (EUR 230/month for permanent residence <80 km from RDU and EUR 800/month for permanent residence >80 km from RDU).

## Data Availability

The data that support the findings of this study are available from the corresponding author upon reasonable request.
